# Protective SARS-CoV-2 Antibody Response in Children With Inflammatory Bowel Disease

**DOI:** 10.3389/fped.2022.815857

**Published:** 2022-02-10

**Authors:** Luca Bosa, Costanza Di Chiara, Paola Gaio, Chiara Cosma, Andrea Padoan, Sandra Cozzani, Giorgio Perilongo, Mario Plebani, Carlo Giaquinto, Daniele Donà, Mara Cananzi

**Affiliations:** ^1^Unit of Pediatric Gastroenterology, Digestive Endoscopy, Hepatology and Care of the Child With Liver Transplantation, Department of Women's and Children's Health, University Hospital of Padova, Padova, Italy; ^2^Pediatric Infectious Diseases, Department of Women's and Children's Health, University Hospital of Padova, Padova, Italy; ^3^Department of Laboratory Medicine, University Hospital of Padova, Padova, Italy; ^4^Department of Medicine-DIMED, Medical School, University of Padova, Padova, Italy; ^5^Department of Women's and Children's Health, University Hospital of Padova, Padova, Italy

**Keywords:** inflammatory bowel disease, children, COVID-19, immunological response, neutralizing antibodies, pediatric, SARS-CoV-2, serology

## Abstract

**Background:**

To date, there's no evidence of an increased risk of SARS-CoV-2 infection or more severe COVID-19 in patients with inflammatory bowel disease (IBD). However, whether COVID-19 alters the clinical course of IBD or whether IBD treatment affects the immunological response to SARS-CoV-2 is still under investigation, especially in children.

**Aim:**

To assess the serological response to SARS-CoV-2 in children with IBD, and to evaluate the impact of COVID-19 on the clinical course of IBD.

**Material and Methods:**

This prospective study enrolled children (0–18 years) followed-up at the University Hospital of Padova for IBD, who acquired a confirmed SARS-CoV-2 infection between 02.2020 and 02.2021. The anti-SARS-CoV-2 S-RBD IgG titer was evaluated at 3 months after infection and compared to that of a control group of healthy children matched for age, sex, and COVID-19 severity.

**Results:**

Twelve children with IBD (*M* = 5; median age 14 years) contracted COVID-19 during the study period. 11/12 patients were under immunomodulatory treatment (4/12 steroids; 6/12 azathioprine; 3/12 anti-TNFs; 2 vedolizumab; 1 ustekinumab). SARS-CoV-2 infection remained asymptomatic in 4/12 children and caused mild COVID-19 in the remaining 8. Mean anti-SARS-CoV-2 IgG S-RBD titer was similar between IBD patients and controls (27.3 ± 43.8 vs. 36.8 ± 35.3 kAU/L, *p* = ns). No children experienced IBD flares nor required gastroenterological support during the infection period.

**Discussion:**

Children with IBD can mount a protective humoral response against SARS-CoV-2, which is comparable to that of their healthy peers regardless of ongoing immunomodulatory treatment. This study also supports the favorable course of PIBD during COVID-19 and vice-versa.

## Introduction

Coronavirus Disease 2019 (COVID-19), caused by severe acute respiratory syndrome coronavirus 2 (SARS-CoV-2), has spread globally, evolving into a pandemic and a serious public health threat across the world (1). When compared to adults, children and adolescents have a mild COVID-19 course with a good prognosis (2). However, a small percentage of pediatric subjects experience life-threatening complications from SARS-CoV-2 infection either in the form of severe or critical COVID-19 (3) or in the form of a severe hyperinflammatory condition, known as multisystem inflammatory syndrome in children (MIS-C) (4).

From the beginning of the SARS-CoV-2 emergency, special attention has been reserved to immunocompromised subjects, including patients treated for inflammatory bowel disease (IBD) (5). Despite concerns that patients with IBD could be more susceptible to COVID-19, there is to date no evidence of an increased risk of SARS-CoV-2 infection or development of more severe COVID-19 disease in this patient group compared to the general population, regardless of ongoing immunosuppressive therapy (6–8). However, whether immunomodulatory treatment in IBD affects the degree and the duration of the antibody response to SARS-CoV-2 infection (and/or vaccination) is still under investigation, especially in children (9, 10). The induction of protective immunity to SARS-CoV-2 is critical to contain viral replication in individual subjects, suppress viral transmission across the population, and prevent the emergence of novel viral variants (11, 12). Also, it remains to be determined whether COVID-19 affects the clinical course of IBD. The documented intestinal tropism of the virus, combined with the onset of gastrointestinal symptoms and the high fecal calprotectin levels observed in COVID-19 patients, has led to the hypothesis that SARS-CoV-2 infection could trigger bowel inflammation in subjects with IBD (5, 13, 14). Unfortunately, the overlap between the clinical manifestations of active IBD and the gastrointestinal symptoms of COVID-19 make it challenging to prove or disprove the above speculation during the acute phase of COVID-19 (15).

In the attempt to investigate the consequences of SARS-CoV-2 infection in IBD and vice-versa, we performed a prospective study aiming to primarily assess the serological response to SARS-CoV-2 in a cohort of children with IBD and confirmed COVID-19 and, secondarily, to evaluate the impact of COVID-19 on the clinical course of pediatric IBD (PIBD).

## Materials and Methods

### Study Design and Population

A single-center, prospective, observational study was conducted on pediatric patients (aged 0–18 years old at February 2020) with a diagnosis of Crohn's disease (CD), ulcerative colitis (UC), or unclassified inflammatory bowel disease (IBDU) followed at the Department of Women's and Children's Health (W&CHD) of the University Hospital of Padova, a tertiary referral pediatric hospital and a regional center for PIBD in the North-East of Italy.

All the subjects of the cohort who acquired a SARS-CoV-2 infection from the February 21, 2020 (date of the first case of COVID-19 diagnosed in Italy) to February 28, 2021 were enrolled in the study after obtainment of the informed consent from the parents or legally authorized representatives.

### Case Definition

Patients were included in the study if satisfying the WHO definition of confirmed SARS-CoV-2 infection following either a positive Nucleic Acid Amplification Test (NAAT) or a positive SARS-CoV-2 antigen-rapid diagnostic test (RDT) and contact with a probable or confirmed COVID-19 case or cluster (16).

Patients were also included in the study if satisfying the WHO criteria for probable SARS-CoV-2 infection (i.e., a patient who meets COVID-19 clinical criteria and is a contact of a probable or confirmed COVID-19 case or cluster) in adjunct to a positive serological test obtained after the resolution of symptoms (16, 17).

In confirmed COVID-19 cases, SARS-CoV-2 infection was dated either from the first positive diagnostic test (NAAT or RDT) or from the onset of symptoms. In serologically identified COVID-19 patients, SARS-CoV-2 infection was dated back to the onset of symptoms after the close contact with a probable or confirmed COVID-19 case or cluster.

The severity of COVID-19 was scored as mild, moderate, severe, or critical following the WHO classification based on clinical features, laboratory testing, and chest radiograph imaging (when available). Severe COVID-19 is defined by oxygen saturation <90% on room air or by signs of severe respiratory distress. Critical COVID-19 is defined by the criteria for acute respiratory distress syndrome (ARDS), sepsis, septic shock, or other conditions that would require the provision of life-sustaining therapies such as mechanical ventilation (invasive or non-invasive) or vasopressor therapy, or by a disease resulting in death (18).

### Data Collection

PIBD patients with SARS-CoV-2 infection were actively identified by in-person or phone interviews with the parents, inquiring about SARS-CoV-2 testing, COVID-19 diagnosis, COVID-19 symptoms, close contacts with probable or confirmed COVID-19 cases, and quarantine assignments.

All patients experiencing the acute onset of gastroenterological symptoms in the context of IBD remission or a worsening of gastroenterological symptoms in comparison to baseline underwent a NAAT or a RDT. Moreover, a surveillance NAAT was performed in all PIBD patients at the time of biologic drug infusions or endoscopic check-ups, and in case of hospital admission or emergency department visits.

A standardized questionnaire was used to collect all data related to SARS-CoV-2 infection, including diagnostic modalities, date of diagnosis, source and duration of infection, clinical manifestations, duration of symptoms, clinical outcome, infection management, need for hospital admission or anti-viral treatment. The impact of COVID-19 on the course and the management of IBD was also investigated, inquiring each patient for disease activity before, at the time and after SARS-CoV-2 infection [remission was defined by a PCDAI (19) score <10 for CD, and a PUCAI score (20) <10 for UC and IBDU], fecal calprotectin levels before and after SARS-CoV-2 infection (the cu*t-*off value of 250–300 μg/g was considered predictive of mucosal inflammation) (21), modifications of IBD treatment during the infection, and possible variations on scheduled hospital admissions, endoscopic procedures or outpatient visits.

Demographic data (age, gender, ethnicity) and all the information concerning the patients' medical history, with particular regard to IBD [date of diagnosis, disease type (22), extent, and disease behavior (23) and treatment], were obtained through clinical file revision.

Collected data were entered into a password-protected Excel database and analyzed anonymously.

At 3 months from the SARS-CoV-2 infection onset, all IBD patients received a gastroenterology and infectious diseases clinical evaluation along with a blood sample collection for SARS-CoV-2 serology testing.

### Serological Assay

The serological response to SARS-CoV-2 infection was evaluated in PIBD patients with confirmed COVID-19 at 3 months after infection employing a commercially available chemiluminescent immunoassay (CLIA) measuring the IgG antibody titer against the receptor-binding domain (RBD) of the spike (S) protein in human serum (anti-SARS-CoV-2 S-RBD IgG; Snibe Diagnostics, New Industries Biomedical Engineering Co., Shenzhen, China) (2, 3). According to the manufacturer's instructions, all analyses were performed using a magnetic microbead separation technology (MAGLUMI™2000 Plus, Snibe Diagnostics). Results were expressed in arbitrary units. The electrochemiluminescence signal from the reaction product was considered positive when ≥1.0 kAU/L in accordance with the cu*t-*off recommended by the manufacturer (24).

### Control Group and Case-Control Study

The anti-SARS-CoV-2 S-RBD IgG titles of IBD patients in our cohort were matched 1:4 for age, sex, and COVID-19 severity with a control group of healthy children convalescent after SARS-CoV-2 infection. This control group was recruited from the COVID-19 Family Cluster Follow-up Outpatient Clinic (CovFC) program of the University Hospital of Padova, which included the measurement of anti-SARS-CoV-2 S-RBD IgG in healthy children (0–18 years old) at 3 months after SARS-CoV-2 infection (25, 26). The Institutional Review Board approved the CovFC study protocol, and parents or legally authorized representatives of the children included in the control group provided written informed consent to use clinical data for research purposes.

### Statistical Analysis

Descriptive statistics were used to summarize the basic demographic and clinical characteristics of the study population. Data were summarized as mean ± standard deviation or median and interquartile range for quantitative variables, as counts and percentages for categorical variables. The normality of quantitative variables was checked with the Shapiro-Wilk test. Quantitative variables were compared across groups with independent *t-*test and categorical variables with χ2 or Fisher exact test, as appropriate. Significance was set at *p-*value < 0.05. Analyses were performed using SPSS 23.0 (IBM Corporation, Armonk, NY).

## Results

### Pediatric IBD Patients With SARS-CoV-2 Infection

Eighty-four pediatric patients with IBD (median age 14 years, range 1–18 years, IQR 12.25–17 years) were in follow-up at the W&CHD of the University Hospital of Padova between the 21st of February 2020 and the 28th of February 2021 (Table S1). None of them received any anti-SARS-CoV-2 vaccination as, in Italy, no vaccine against COVID-19 was available for pediatric patients before March 2021 (27). Twelve children (*M* = 5; median age 15 years, range 12–18 years, IQR 14–17 years) contracted a SARS-CoV-2 infection during the study period and were enrolled in the study. Nine out of these twelve children were affected by CD, one by UC and two by IBDU. At the time of SARS-CoV-2 infection, half of the patients (6/12) were on azathioprine treatment, and one-third (4/12) were on steroids. Three children were treated with anti-TNF agents (2 with adalimumab and one with infliximab), two with vedolizumab, and one with ustekinumab (Table 1). In all patients, the IBD treatment was continued without interruption for the entire duration of COVID-19.

**Table 1:** Demographics and clinical characteristics of SARS-CoV-2 cases within our pediatric IBD cohort.

**IBD case**	**Age at SARS-CoV-2 infection (years)**	**Sex**	**Ethnicity**	**IBD type**	**IBD therapy**	**IBD activity (PCDAI/PUCAI) before/after COVID-19**		**Fecal calprotectin before/after COVID-19 (ug/g)**		**Relevant comorbidities**	**Reason for testing**	**Contact setting**	**COVID-19 severity**	**COVID-19 symptoms**	Viral clearance (days)^*^	**COVID-19 outcome**	**SARS-CoV-2 S-RBD IgG (KuA/L)**	**Timing of serology (days)^**§**^**	**Impact on IBD management**
1	12	M	Caucasian	IBDU	None	Remission (0)	Remission (0)	>2,100	1,924	PSC, Hashimoto thyroiditis	Close contact	Health care	Mild	Fever, fatigue, headache, hypogeusia, sore throat, nausea	9	Complete recovery	145	156	Delayed endoscopy
2	13	F	Caucasian	CD	UST, PDN	Remission (5)	Remission (7.5)	>2,100	441	PSC, NAFLD	Close contact	Household	Asymptomatic	None	16	Complete recovery	20,03	159	Delayed visit and biologic drug administration
3	14	F	Arab-Berber	CD	ADA, PEN	Mild (22.5)	Mild (22.5)	>2,100	603	Sacroileitis	Close contact	Household	Asymptomatic	None	11	Complete recovery	4,7	172	None
4	14	F	Arab-Berber	CD	AZA, PEN	Remission (0)	Remission (7.5)	137	<40	None	Close contact	Household	Asymptomatic	None	11	Complete recovery	36,5	172	None
5	14	M	Caucasian	IBDU	AZA, PDN	Remission (0)	Remission (0)	28	79	ASC/AIH	COVID-19 symptoms	Community	Mild	Headache, anosmia, ageusia, rhinitis, sore throat	10	Complete recovery	21.4	89	None
6	14	M	Caucasian	CD	VDZ, PDN	Mild (20)	Remission (0)	>2,100	126	Arthritis, partial IgA deficiency	Close contact	Community	Mild	Fatigue, cough	33	Complete recovery	3,505	98	None
7	16	M	Caucasian	CD	AZA	Remission (0)	Remission (0)	474	162	None	Close contact	Household	Mild	Headache, ageusia, rhinitis	10	Complete recovery	2,5	112	Delayed visit
8	16	F	Caucasian	RCU	IFX, AZA, 5-ASA	Remission (0)	Remission (5)	>2,100	>2,100	None	COVID-19 symptoms	Household	Mild	Fever, fatigue, headache, hyposmia, cough, rhinitis, diarrhea	34	Complete recovery	2,794	109	Delayed visit and biologic drug administration
9	17	F	Caucasian	CD	AZA	Remission (0)	Remission (0)	28	249	None	Close contact	Household	Mild	Fever, cough, sore throat, abdominal pain, diarrhea	NA	Complete recovery	82,2	76	None
10	17	M	Caucasian	CD	VDZ, PDN, MTX	Remission (7.5)	Remission (0)	2,075	1,117	HLA-B27 negative spondyloarthritis	Close contact	Household	Mild	Cough, rhinitis	27	Complete recovery	1,74	128	Delayed visit and biologic drug administration
11	18	F	Caucasian	CD	ADA, PEN	Remission (0)	Remission (0)	310	461	None	COVID-19 symptoms	Unknown	Mild	Fatigue, dysgeusia	NA	Complete recovery	5,5	149	None
12	18	F	Caucasian	CD	AZA	Remission (0)	Remission (5)	300	NA	None	Screening	Unknown	Asymptomatic	None	11	Complete recovery	1,681	132	Delayed endoscopy

*Close contact (of a confirmed COVID-19 case) refers to high-risk exposure, according to the European Center for Disease Prevention and Control (ECDC)*.

* *Viral clearance reflects the time from the first positive diagnostic test to the first negative test*.

§ *Timing of serology refers to the number of days elapsed from disease onset (the day when the symptoms started, or of the first positive nasopharyngeal swab in asymptomatic cases) to serological test*.

*5-ASA, Mesalazine; ADA, Adalimumab; AIH, autoimmune hepatitis; ASC, autoimmune sclerosing cholangitis; AZA, Azathioprine; CD, Crohn's disease; F, female; IBD; inflammatory bowel disease; IBDU, inflammatory bowel disease unclassified; IFX, Infliximab; M, male; MTX, methotrexate; N/A, not available; NAFLD, Nonalcoholic Fatty Liver Disease; PCDAI, Pediatric Crohn's Disease Activity Index; PDN, prednisone; PEN, partial enteral nutrition; PSC, primary sclerosing cholangitis; PUCAI, Pediatric Ulcerative Colitis Activity Index; UC, Ulcerative Colitis; UST, Ustekinumab; VDZ, Vedolizumab; WHO, World Health Organization*.

### Clinical Course of SARS-CoV-2 Infection in Children With IBD

The diagnosis of SARS-CoV-2 infection was based on a positive NAAT in eleven children (91.7%) and on a positive serological test in a single child who experienced fever during the cohabitation with a confirmed COVID-19 relative (8.3%). Ten out of twelve patients (83.3%) contracted the infection after close contact with a COVID-19 subject, respectively in the household (*n* = 7), healthcare (*n* = 1), or community (*n* = 2) setting, while the source of infection was unknown in two patients (Table 1).

SARS-CoV-2 infection remained asymptomatic in four out of twelve children and caused a mild COVID-19 in the remaining eight. The most common complaints were constituted by fatigue, headache, and upper respiratory symptoms (cold, cough, sore throat). Gastroenterological symptoms occurred in three patients: nausea in one and acute diarrhea in two. Apart from the sporadic use of antipyretic or anti-inflammatory drugs, none of the symptomatic patients required anti-viral treatment or hospitalization for COVID-19, and all promptly recovered without sequelae after an average of 5.1 days (SD 3.7) from symptoms onset. The average time from the first positive diagnostic test to the first negative test was 17.2 days (SD 10.1) (Table 2).

**Table 2:** Demographics, COVID-19 clinical and serological features of pediatric IBD patients vs. healthy children with SARS-CoV-2 infection.

	**Cases**		**Controls**		***p-*value**
	**(*****n** **=*** **12)**		**(*****n** **=*** **48)**		
	**Mean (SD)**	**Median (IQR)**	**Mean (SD)**	**Median (IQR)**	
Age (years)	15.3 (2)	15 (14–17)	17.3 (9.5)	13 (10.3–25.3)	0.169
Male	5	41.7	20	41.7	1.000
	n	%	*n*	%	
COVID-19 WHO classification					1.000
Asymptomatic	4	33.3	15	31.3	
Mild	8	66.7	33	68.8	
Moderate or more	0	0	0	0	
COVID-19 symptom duration (days)	5.1 (3.7)	3 (2.25–9.25)	5.2 (4.1)	5.2 (4.1)	0.972
Viral clearance time (days)	17.2 (10.1)	11 (10–28.5)	15.1 (5.8)	13 (10–18.5)	0.548
Collection time (days from infection)	129.3 (32.8)	130 (100.8–158.3)	129.5 (19.3)	133 (11–143.5)	0.985
IgG title (kAU/L)	27.3 (43.8)	5.1 (2.6–32.7)	31.7 (33)	24.5 (12.7–39.5)	0.700

### Serological Response to SARS-CoV-2 Infection in Children With IBD in Comparison to a Control Group of Healthy Children

The immunological response to SARS-CoV-2 infection of our cohort of twelve PIBD patients was compared to a control group of forty-eight healthy children convalescent after COVID-19. The control group was similar according to age, sex, COVID-19 severity, duration of symptoms, and the time between the initial positive to the first negative diagnostic test (Table 2). Each IBD patient was combined to 4 selected controls matched for age, sex, and COVID-19 severity.

**Figure 1:**
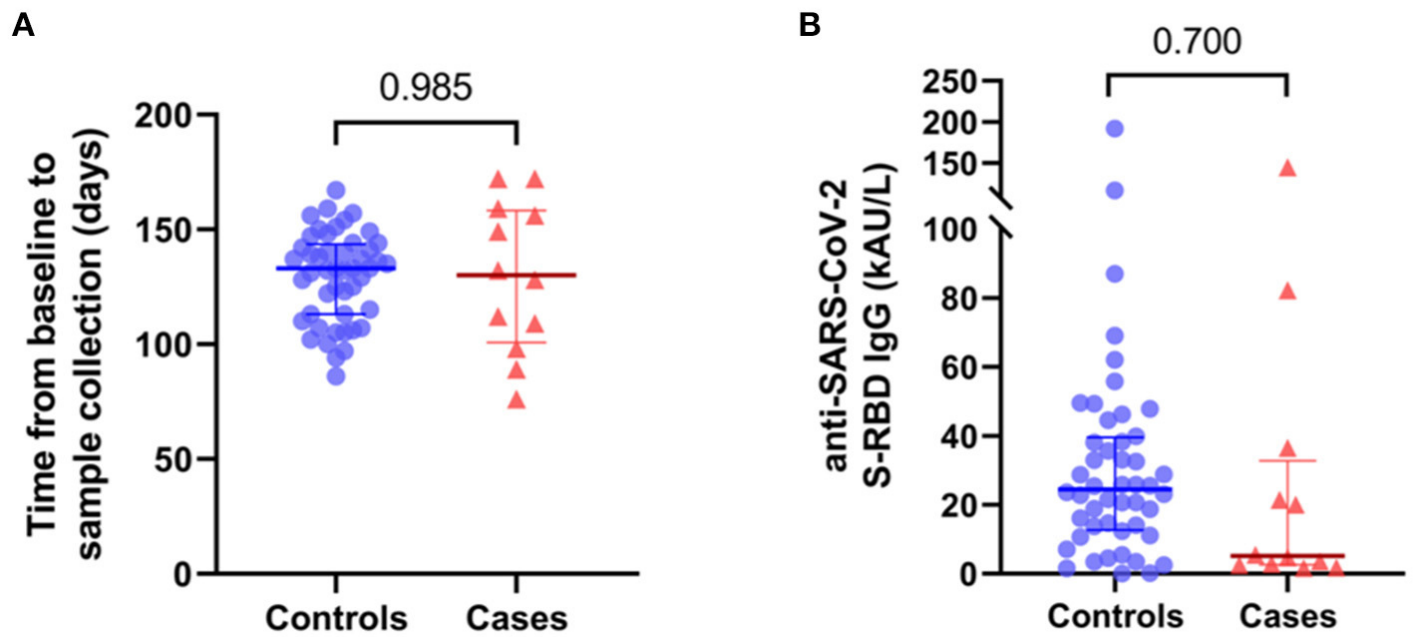
**(A)** Time(days) from baseline to sera collection of controls and cases. **(B)** IgG S-RBD title (kAU/L) of controls and cases. Median and IQR are reported.

SARS-CoV-2 serology was evaluated in both groups 3 months after infection (129 ± 31 days vs. 115 ± 21 days from infection in cases and controls, respectively; *p* = 0.985; Figure 1A and Table 2). The mean anti-SARS-CoV-2 IgG S-RBD title was similar between IBD patients and healthy children (27.3 ± 43.8 kAU/L vs. 36.8 ± 35.3 kAU/L, *p* = 0.451; Figure 1B and Table 2). Since cases with outlier levels of IgG were present (i.e., patients 1 and 9), outlier controls were also included. No clinical, demographic, and comorbidity differences were reported between outliers and other subjects.

### Impact of SARS-CoV-2 Infection on IBD Clinical Course and Management

At the time of SARS-CoV-2 infection, IBD was in remission in ten out of twelve patients, and mild CD symptoms were present in two children. Of these two, one had a persistent mildly active CD after COVID-19 recovery, while all the other children were in clinical remission following infection. No children experienced an IBD flare nor required gastroenterological support during the infection period. Fecal calprotectin, measured at 3 months after SARS-CoV-2 infection and compared to pre-COVID-19 levels, did not significantly worsen in any patient but remained stable or improved in six and four subjects, respectively. No patient had the IBD treatment interrupted, but three patients experienced a delay in biologic drug infusions due to COVID-19 confinement measures. Similarly, scheduled endoscopic reassessments and outpatient visits were postponed in two and four patients, respectively (Table 1).

## Discussion

Our prospective study shows that children with PIBD under treatment with immunomodulatory agents can mount a humoral immunological response against SARS-CoV-2 comparable to that of a control group of healthy subjects matched for age, sex and COVID-19 severity. Our data also provide further evidence for the overall benign course of COVID-19 in PIBD patients regardless of immunosuppressive therapy, and show that SARS-CoV-2 infection does not negatively influence the course of IBD in children previously co-affected with COVID-19.

Specifically, in our cohort of children with IBD, we described the clinical course of COVID-19 in twelve children, most of whom were receiving an immunomodulatory treatment either as a monotherapy or a drug combination. In all cases, the ongoing IBD treatment was continued without interruption for the entire COVID-19 duration according to currently available recommendations (28–30). All of our patients presented an asymptomatic infection or a mild COVID-19, and none required hospitalization. These results align with the most extensive study on PIBD (including 209 patients from the SECURE-IBD registry and the COVID-19 database of the Pediatric IBD Porto group), which reported a low risk of severe COVID-19 even in children receiving biologics and/or other immune-suppressive therapies (8). Moreover, corticosteroid therapy, assumed by a third of our patients, was not associated with a worse COVID-19 outcome, differently from that reported in some adult IBD studies (5, 7, 8, 31–33). Gastrointestinal symptoms arose in 25% of the patients, similarly to that reported in the above-mentioned international cohort of children with IBD (8). Diarrhea occurred in 17% of our patients, a percentage comparable to that of the other reported pediatric and adult IBD patients with COVID-19 (13 and 20%, respectively), but higher than that reported in the non-IBD population (4% and 7–10% in children and adults, respectively) (2, 5, 8, 34).

In adult and pediatric immunocompetent subjects, the natural infection with SARS-CoV-2 initiates a humoral immune response that produces antibodies against specific viral antigens (21, 26, 35–37). Among the latter, antibodies against the receptor-binding domain (RBD) within the S protein, which binds the cellular receptor for viral entry (ACE2), constitute the primary source of neutralizing antibodies (i.e., able to inhibit viral replication *in vitro*) and are considered a reliable measure to assess anti-viral immunity (24, 38, 39). Indeed, the appearance of neutralizing antibodies is associated with viral clearance and their absence with an increased risk of fatal outcomes (35, 39). The kinetic of antibody response shows large interindividual variability and varies significantly depending on multiple factors such as patient age, sex and immune status, COVID-19 severity, and testing system (17, 26, 37, 38, 40–44).

In our cohort of PIBD patients, all children with COVID-19 developed anti-SARS-CoV-2 S-RBD antibodies. Moreover, independently from the ongoing IBD treatment, the titer of anti-spike antibodies resulted similar to a control population of healthy children matched for age, sex, and COVID-19 severity. These results are in agreement with the observation that immunocompromised adult IBD patients with COVID-19 can develop a serologic response comparable to that of age-matched healthy controls (45). They are also in line with a recent study reporting a high rate of seroconversion (85%) in immunocompromised PIBD patients (*n* = 12, median age 10 years, range 2–17 years) at a median time of 8 weeks after SARS-CoV-2 infection (46). Our results differ, however, from the study of Dailey et al. who reported lower titers of anti-SARS-CoV-2 S-RBD antibodies in pediatric and young adult IBD patients (*n* = 44, median age 18 years, range 11–26 years) in comparison to a control group of non-IBD children and adults (47). The different outcome may be related to the diverse characteristics of control groups: in the study of Dailey's et al. control subjects consisted of non-IBD children hospitalized with SARS-CoV-2 infection and of non-IBD adults with mild to moderate COVID-19, while we compared PIBD patients 1:4 to healthy children matched for age, sex, and COVID-19 severity.

Contrarily to adult IBD reports (9) but similarly to another PIBD study (47), we did not observe different levels of anti-SARS-CoV-2 antibodies between children treated with anti-TNFs and children receiving other biologics or immunomodulatory drugs, probably due to the small size of our patient population. We also did not observe serological differences relative to COVID-19 severity since all patients in our cohort had a mild form of disease. Notably, children with high antibody titers (i.e., IgG outliers) were observed in both the IBD and the control group, likely reflecting immunological inter-individual variability.

In immunocompetent subjects, anti-SARS-CoV-2 (neutralizing) IgG antibodies are detectable for several months in most persons (17, 26, 39). How long these antibodies persist after infection in exogenously immunocompromised subjects is still under investigation (36). In our cohort of children with IBD, we detected anti-SARS-CoV-2 S-RBD IgG at 3 months after COVID-19, consistently with the observation of detectable anti-SARS-CoV-2 antibodies up to 2 months after infection in adult and pediatric IBD patients treated with biologics (47, 48). We did not investigate the kinetic of antibody response over time. Although future studies are needed to assess the persistence of anti-S RDB antibodies in the long term, the similar viral clearance time (i.e., time between the initial positive to the first negative diagnostic test) and the comparable titer of neutralizing SARS-CoV-2 S-RBD antibodies allow hypothesizing a similar seroconversion time among children with IBD and healthy pediatric controls (24).

To date, it remains uncertain whether SARS-CoV-2 infection affects the clinical course of IBD, especially in children. As for our cohort, none of our twelve COVID-19 patients experienced a disease flare or a worsening of disease activity after SARS-CoV-2 infection. Likewise, no significant elevation of fecal calprotectin was documented (neither in those patients experiencing gastrointestinal symptoms during the acute phase of COVID-19), but a significant improvement was observed in one-third of the patients. Moreover, none of the PIBD patients experiencing a disease flare during the study period tested positive for SARS-CoV-2. These results are in agreement with those of a single, recent, study reporting that no children with IBD (*n* = 44) suffered a disease flare following SARS-CoV-2 infection (47).

Nevertheless, in our cohort of PIBD children, COVID-19 caused several forced delays in IBD management due to the containment measures required to avoid SARS-CoV-2 transmission by positive patients. Two children experienced a delay of planned biologic drug infusions, and another one in the initiation of biologic treatment. Similarly, scheduled endoscopic reassessment and outpatient visits were postponed in two and four patients, respectively. As previously reported (49–51), the SARS-CoV-2 pandemic is strongly challenging the diagnostic and therapeutic management of IBD across the globe. Further studies will be needed to evaluate the long-term impact of the pandemic on the course of PIBD.

Our study has several limitations. First of all, the small number of PIBD patients with a confirmed SARS-CoV-2 infection, all adolescents between 12 and 18 years of age, hampers the possibility to draw definitive conclusions. Secondly, the therapeutic heterogeneity among enrolled patients does not allow to investigate the potential effect of pharmacological treatments on the serological response against SARS-CoV-2 or to perform any sub-group analysis. Lastly, the strict adherence to the WHO definition of confirmed SARS-CoV-2 infection may have resulted in the lack of recognition of asymptomatic COVID-19 patients and in an underestimation of eligible patients.

Despite being a small monocentric study, the present work has several strengths. To the best of our knowledge, this is one of the very few studies investigating the immunological response to SARS-CoV-2 and the impact of COVID-19 on IBD course in pediatric IBD patients (46, 47). Only subjects satisfying the WHO case definition of confirmed SARS-CoV-2 infection were enrolled in the study (both in the patient and in the control group). The serologic response to SARS-COV-2 infection was determined based on the titers of anti-S-RBD IgG, the primary source of neutralizing antibodies (24, 38, 39), employing a high performant diagnostic test (24). The control population of non-IBD subjects was comparable to the group of IBD patients for demographic, COVID-19 characteristics, and timing of serological tests.

In conclusion, this prospective study shows that children with IBD can mount a protective humoral response against SARS-CoV-2 and further support for the overall favorable course of COVID-19 in PIBD (and vice-versa) regardless of ongoing immunomodulatory treatment. Further studies are needed to confirm these results in a broader population of children with IBD, to determine the longevity of humoral immunity over time, and to assess the serological response to COVID-19 vaccines in this patient group.

## Data Availability Statement

The original contributions presented in the study are included in the article/Supplementary Material, further inquiries can be directed to the corresponding author/s.

## Ethics Statement

Ethical review and approval was not required for the study on human participants in accordance with the local legislation and institutional requirements. Written informed consent to participate in this study was provided by the participants' legal guardian/next of kin.

## Author Contributions

LB, CD, and MC wrote the manuscript and prepared the figures and which all authors reviewed. LB, PG, and MC provided clinical care, collected and reviewed clinical data of the IBD study cohort. CD, SC, and DD collected and reviewed clinical data of the CovFC study cohort. CC and AP performed all serological analyses. GP, MP, CG, and DD performed a critical review of the findings and participated in the preparation of the manuscript. All authors contributed to the article and approved the submitted version.

## Funding

Our study participates in Orchestra, a three-year international research project aimed at tackling the coronavirus pandemic, which is funded by the European Union's Horizon 2020 research and innovation program (H2020-RIA GA No. 101016167). The views expressed in this paper are the sole responsibility of the author and the Commission is not responsible for any use that may be made of the information it contains.

## Conflict of Interest

The authors declare that the research was conducted in the absence of any commercial or financial relationships that could be construed as a potential conflict of interest.

## Publisher's Note

All claims expressed in this article are solely those of the authors and do not necessarily represent those of their affiliated organizations, or those of the publisher, the editors and the reviewers. Any product that may be evaluated in this article, or claim that may be made by its manufacturer, is not guaranteed or endorsed by the publisher.

## References

[1] Zhu N Zhang D Wang W Li X Yang B Song J. A Novel Coronavirus from Patients with Pneumonia in China, 2019. N Engl J Med. (2020) 382:727–33. 10.1056/NEJMoa2001017 31978945 PMC7092803

[2] Mantovani A Rinaldi E Zusi C Beatrice G Saccomani MD Dalbeni A. Coronavirus disease 2019 (COVID-19) in children and/or adolescents: a meta-analysis. Pediatr Res. (2021) 89:733–7. 10.1038/s41390-020-1015-2 32555539

[3] Badal S Thapa Bajgain K Badal S Thapa R Bajgain BB Santana MJ. Prevalence, clinical characteristics, and outcomes of pediatric COVID-19: a systematic review and meta-analysis. J Clin Virol Off Publ Pan Am Soc Clin Virol. (2021) 135:104715. 10.1016/j.jcv.2020.104715 33348220 PMC7723460

[4] Keshavarz P Yazdanpanah F Azhdari S Kavandi H Nikeghbal P Bazyar A. Coronavirus disease 2019 (COVID-19): A systematic review of 133 Children that presented with Kawasaki-like multisystem inflammatory syndrome. J Med Virol. (2021) 93:5458–73. 10.1002/jmv.27067 33969513 PMC8242327

[5] D'Amico F Danese S Peyrin-Biroulet L. Systematic Review on Inflammatory Bowel Disease Patients With Coronavirus Disease 2019: It Is Time to Take Stock. Clin Gastroenterol Hepatol. (2020) 18:2689–700. 10.1016/j.cgh.2020.08.003 32777550 PMC7831523

[6] Allocca M Chaparro M Gonzalez HA Bosca-Watts MM Palmela C D'Amico F. Patients with Inflammatory Bowel Disease Are Not at Increased Risk of COVID-19: A Large Multinational Cohort Study. J Clin Med. (2020) 9:3533. 10.3390/jcm9113533 33142843 PMC7693947

[7] Burke KE Kochar B Allegretti JR Winter RW Lochhead P Khalili H. Immunosuppressive Therapy and Risk of COVID-19 Infection in Patients with Inflammatory Bowel Diseases. Inflamm Bowel Dis. (2021) 27:155–61. 10.1093/ibd/izaa278 33089863 PMC7665507

[8] Brenner EJ Pigneur B Focht G Zhang X Ungaro RC Colombel JF. Benign evolution of SARS-Cov2 infections in children with inflammatory bowel disease: results from two international databases. Clin Gastroenterol Hepatol. (2021) 19:394–6.e5. 10.1016/j.cgh.2020.10.010 33059040 PMC7550063

[9] Kennedy NA Goodhand JR Bewshea C Nice R Chee D Lin S. Anti-SARS-CoV-2 antibody responses are attenuated in patients with IBD treated with infliximab. Gut. (2021) 70:865–75. 10.1136/gutjnl-2021-324388 33753421

[10] D'Amico F Rabaud C Peyrin-Biroulet L Danese S. SARS-CoV-2 vaccination in IBD: more pros than cons. Nat Rev Gastroenterol Hepatol. (2021) 18:211–3. 10.1038/s41575-021-00420-w 33473178 PMC7816748

[11] Alexander JL Moran GW Gaya DR Raine T Hart A Kennedy NA. SARS-CoV-2 vaccination for patients with inflammatory bowel disease: a British Society of Gastroenterology Inflammatory Bowel Disease section and IBD Clinical Research Group position statement. Lancet Gastroenterol Hepatol. (2021) 6:218–24. 10.1016/S2468-1253(21)00024-8 33508241 PMC7834976

[12] Centers for Disease Control Prevention. SARS-CoV-2 Variant Classifications and Definitions. Available online at: https://www.cdc.gov/coronavirus/2019-ncov/variants/variant-classifications.html (accessed December 01, 2021).

[13] Effenberger M Grabherr F Mayr L Schwaerzler J Nairz M Seifert M. Faecal calprotectin indicates intestinal inflammation in COVID-19. Gut. (2020) 69:1543–4. 10.1136/gutjnl-2020-321388 32312790 PMC7211078

[14] Liu J Li Y Liu Q Yao Q Wang X Zhang H. SARS-CoV-2 cell tropism and multiorgan infection. Cell Discov. (2021) 7:17. 10.1038/s41421-021-00249-2 33758165 PMC7987126

[15] Yang C Xiao SY. COVID-19 and inflammatory bowel disease: a pathophysiological assessment. Biomed Pharmacother. (2021) 135:111233. 10.1016/j.biopha.2021.111233 33433350 PMC7834878

[16] World Health Organization. WHO. COVID-19: Case Definitions: Updated in Public Health Surveillance for COVID-19. World Health Organization. (2020). 10.15557/PiMR.2020.0006.

[17] Centers for Disease Control Prevention. Interim Guidelines for COVID-19 Antibody Testing. Available online at: https://www.cdc.gov/coronavirus/2019-ncov/lab/resources/antibody-tests-guidelines.html (accessed January 24, 2022).

[18] World Health Organization. Clinical Management of COVID-19: Interim Guidance. World Health Organization (2020). 10.15557/PiMR.2020.0004.

[19] Hyams JS Ferry GD Mandel FS Gryboski JD Kibort PM Kirschner BS. Development and validation of a pediatric Crohn's disease activity index. J Pediatr Gastroenterol Nutr. (1991) 12:439–47. 10.1097/00005176-199105000-00005 1678008

[20] Turner D Otley AR Mack D Hyams J de Bruijne J Uusoue K. Development, validation, and evaluation of a pediatric ulcerative colitis activity index: a prospective multicenter study. Gastroenterology. (2007) 133:423–32. 10.1053/j.gastro.2007.05.029 17681163

[21] Rostad CA Chahroudi A Mantus G Lapp SA Teherani M Macoy L. Quantitative SARS-CoV-2 serology in children with Multisystem Inflammatory Syndrome (MIS-C). Pediatrics. (2020) 146:e2020018242. 10.1542/peds.2020-018242 32879033

[22] Levine A Koletzko S Turner D Escher JC Cucchiara S de Ridder L. The ESPGHAN revised porto criteria for the diagnosis of inflammatory bowel disease in children and adolescents. J Pediatr Gastroenterol Nutr. (2013) 58:1. 10.1097/MPG.0000000000000239 24231644

[23] Levine A Griffiths A Markowitz J Wilson DC Turner D Russell RK. Pediatric modification of the Montreal classification for inflammatory bowel disease: the Paris classification. Inflamm Bowel Dis. (2011) 17:1314–21. 10.1002/ibd.21493 21560194

[24] Padoan A Bonfante F Cosma C Di Chiara C Sciacovelli L Pagliari M. Analytical and clinical performances of a SARS-CoV-2 S-RBD IgG assay: comparison with neutralization titers. Clin Chem Lab Med. (2021) 59:1444–52. 10.1101/2021.03.10.21253260 33855843

[25] Sirico D Di Chiara C Costenaro P Bonfante F Cozzani S Plebani M. Left ventricular longitudinal strain alterations in asymptomatic or mildly symptomatic paediatric patients with SARS-CoV-2 infection. Eur Hear journal Cardiovasc Imag. (2021) 5:jeab127 10.1093/ehjci/jeaa356.167 34219155

[26] Bonfante F Costenaro P Cantarutti A Di Chiara C Bortolami A Petrara MR. Mild SARS-CoV-2 infections and neutralizing antibody titers. Pediatrics. (2021) 148:e2021052173. 10.1542/peds.2021-052173 34158312

[27] Italian Ministry of Health. National Strategic Vaccine Plan for the prevention of SARS-CoV-2 infections. Available from: https://www.trovanorme.salute.gov.it/norme/dettaglioAtto?id=79430 (accessed December 03, 2021)..

[28] Arrigo S Alvisi P Banzato C Bramuzzo M Civitelli F Corsello A. Management of paediatric IBD after the peak of COVID-19 pandemic in Italy: A position paper on behalf of the SIGENP IBD working group. Digest Liver Dis. (2021) 53:183–9. 10.1016/j.dld.2020.10.024 33132063 PMC7580561

[29] Kennedy NA Jones GR Lamb CA Appleby R Arnott I Beattie RM. British Society of Gastroenterology guidance for management of inflammatory bowel disease during the COVID-19 pandemic. Gut. (2020) 69:984–90. 10.1136/gutjnl-2020-321244 32303607 PMC7211081

[30] Turner D Huang Y Martín-de-Carpi J Aloi M Focht G Kang B. COVID-19 and Paediatric Inflammatory Bowel Diseases: Global Experience and Provisional Guidance (March 2020) from the Paediatric IBD Porto group of ESPGHAN. J Pediatr Gastroenterol Nutr. (2020) 70:727–33. 10.1097/MPG.0000000000002729 32443020 PMC7273950

[31] Brenner EJ Ungaro RC Gearry RB Kaplan GG Kissous-Hunt M Lewis JD. Corticosteroids, but not TNF antagonists, are associated with adverse COVID-19 outcomes in patients with inflammatory bowel diseases: results from an international registry. Gastroenterology. (2020) 159:481–91.e3. 10.1053/j.gastro.2020.05.032 32425234 PMC7233252

[32] Bezzio C Saibeni S Variola A Allocca M Massari A Gerardi V. Outcomes of COVID-19 in 79 patients with IBD in Italy: An IG-IBD study. Gut. (2020) 69:1213–7. 10.1136/gutjnl-2020-321411 32354990

[33] Lukin DJ Kumar A Hajifathalian K Sharaiha RZ Scherl EJ Longman RS. Baseline disease activity and steroid therapy stratify risk of COVID-19 in patients with inflammatory bowel disease. Gastroenterology. (2020) 159:1541–4.e2. 10.1053/j.gastro.2020.05.066 32479824 PMC7256492

[34] Mao R Qiu Y He J-S Tan J-Y Li X-H Liang J. Manifestations and prognosis of gastrointestinal and liver involvement in patients with COVID-19: a systematic review and meta-analysis. lancet Gastroenterol Hepatol. (2020) 5:667–78. 10.1016/S2468-1253(20)30126-6 32405603 PMC7217643

[35] Chvatal-Medina M Mendez-Cortina Y Patiño PJ Velilla PA Rugeles MT. Antibody responses in COVID-19: a review. Front Immunol. (2021) 12:633184. 10.3389/fimmu.2021.633184 33936045 PMC8081880

[36] Huang AT Garcia-Carreras B Hitchings MDT Yang B Katzelnick LC Rattigan SM. A systematic review of antibody mediated immunity to coronaviruses: kinetics, correlates of protection, and association with severity. Nat Commun. (2020) 11:4704. 10.1038/s41467-020-18450-4 32943637 PMC7499300

[37] Weisberg SP Connors TJ Zhu Y Baldwin MR Lin W-H Wontakal S. Distinct antibody responses to SARS-CoV-2 in children and adults across the COVID-19 clinical spectrum. Nat Immunol. (2021) 22:25–31. 10.1038/s41590-020-00826-9 33154590 PMC8136619

[38] Galipeau Y Greig M Liu G Driedger M Langlois M-A. Humoral Responses and Serological Assays in SARS-CoV-2 Infections. Front Immunol. (2020) 11:610688. 10.3389/fimmu.2020.610688 33391281 PMC7775512

[39] Dispinseri S Secchi M Pirillo MF Tolazzi M Borghi M Brigatti C. Neutralizing antibody responses to SARS-CoV-2 in symptomatic COVID-19 is persistent and critical for survival. Nat Commun. (2021) 12:2670. 10.1038/s41467-021-22958-8 33976165 PMC8113594

[40] Anderson EM Diorio C Goodwin EC McNerney KO Weirick ME Gouma S. Severe acute respiratory syndrome-coronavirus-2 (SARS-CoV-2) antibody responses in children with multisystem inflammatory syndrome in children (MIS-C) and mild and severe coronavirus disease 2019 (COVID-19). J Pediatric Infect Dis Soc. (2021) 10:669–73. 10.1093/jpids/piaa161 33263756 PMC7799010

[41] Petersen LR Sami S Vuong N Pathela P Weiss D Morgenthau BM. Lack of antibodies to SARS-CoV-2 in a large cohort of previously infected persons. Clin Infect Dis. (2020) 73:e3066–73. 10.1093/cid/ciaa1685 33147319 PMC7665429

[42] Kaufman HW Chen Z Meyer WA 3rd Wohlgemuth JG. Insights from patterns of SARS-CoV-2 immunoglobulin G serology test results from a National Clinical Laboratory, United States, March-July 2020. Popul Health Manag. (2021) 24:S35–42. 10.1089/pop.2020.0256 33216694 PMC7875137

[43] Stainer A Amati F Suigo G Simonetta E Gramegna A Voza A. COVID-19 in immunocompromised patients: a systematic review. Semin Respir Crit Care Med. (2021) 42:839–58. 10.1055/s-0041-1740110 34918325

[44] Connelly JA Chong H Esbenshade AJ Frame D Failing C Secord E. Impact of COVID-19 on pediatric immunocompromised patients. Pediatr Clin North Am. (2021) 68:1029. 10.1016/j.pcl.2021.05.007 34538297 PMC8149202

[45] Berte' R Mazza S Stefanucci MR Noviello D Costa S Ciafardini C. Seroprevalence of SARS-CoV2 in IBD patients treated with biologic therapy. J Crohn's Colitis. (2020) 1–15. 10.1093/ecco-jcc/jjaa237 PMC7717179 33211810

[46] Ruan W Nguyen H Wyatt A Ihekweazu F Vartabedian BS Karam L. High seroconversion rate against severe acute respiratory syndrome coronavirus 2 in symptomatic pediatric inflammatory bowel disease patients. J Pediatr Gastroenterol Nutr. (2021) 73:363–6. 10.1097/MPG.0000000000003211 34173793 PMC8373382

[47] Dailey J Kozhaya L Dogan M Hopkins D Lapin B Herbst K. Antibody responses to SARS-CoV-2 after infection or vaccination in children and young adults with inflammatory bowel disease. Inflamm Bowel Dis. (2021) 16:1–8. 10.1093/ibd/izab207 34528661 PMC8499989

[48] Scharrer S Kutschera M Weseslindtner L Primas C Vogelsang H. Humoral response to COVID-19 infection in immunosuppressed patients with inflammatory bowel disease. Eur J Gastroenterol Hepatol. (2021) 33:443–7. 10.1097/MEG.0000000000002094 33522752 PMC7846249

[49] Arrigo S Alvisi P Banzato C Bramuzzo M Celano R Civitelli F. Impact of COVID-19 pandemic on the management of paediatric inflammatory bowel disease: an Italian multicentre study on behalf of the SIGENP IBD Group. Dig Liver Dis. (2021) 53:283–8. 10.1016/j.dld.2020.12.011 33388247 PMC7832380

[50] Ashton JJ Kammermeier J Spray C Russell RK Hansen R Howarth LJ. Impact of COVID-19 on diagnosis and management of paediatric inflammatory bowel disease during lockdown: a UK nationwide study. Arch Dis Child. (2020) 105:1186–91. 10.1136/archdischild-2020-319751 32732316

[51] Maclean A Ashton JJ Garrick V Beattie RM Hansen R. Impact of COVID-19 on the diagnosis, assessment and management of children with inflammatory bowel disease in the UK: implications for practice. BMJ Paediatr open. (2020) 4:e000786. 10.1136/bmjpo-2020-000786 34192173 PMC7549416

