# Supplementary material for: Protective SARS-CoV-2 Antibody Response in Children With Inflammatory Bowel Disease
Source: Front Pediatr. 2022 Feb 10;10:815857. doi: 10.3389/fped.2022.815857 (PMC8866952; doi:10.3389/fped.2022.815857)
Supplement: Supplementary file 1 [file Table_1.DOCX]

**Supplementary Table S1.** Demographic features, IBD diagnosis and ongoing IBD treatment of the pediatric IBD patients followed at the W&CHD of the University Hospital of Padova during the study period.

|  | **Pediatric IBD patients** | **Pediatric IBD patients with a confirmed**  **SARS-CoV-2 infection** | **Pediatric IBD patients without a confirmed**  **SARS-CoV-2 infection** |
| --- | --- | --- | --- |
| **Number** | 84 | 12 | 72 |
| **Mean age ± SD, years** | 14,0 ± 3,5 | 15,4 ± 1,8 | 13,7 ± 3,6 |
| **Sex, n (%)**  M  F | 40 (48)  44 (52) | 5 (42)  7 (58) | 35 (49)  37 (51) |
| **Ethnicity**  Caucasian  Arab-Berber  African  Bengali  Chinese | 73  7  2  1  1 | 10  2  0  0  0 | 63  5  2  1  1 |
| **Diagnosis, n (%)**  CD  UC  IBDU | 49 (58)  21 (25)  14 (17) | 9 (75)  2 (17)  1 (8) | 40 (56)  19 (26)  13 (18) |
| **IBD therapy**  5-ASA  AZA  MTX  Systemic steroids (any dosage)  Budesonide  Tacrolimus  MMF  **Biologics**  IFX  ADA  UST  VDZ  **Combination therapy**  IFX+AZA  ADA+AZA | 31  46  2  15  1  3  1  19  14  5  4  14  8 | 1  6  0  4  1  0  0  1  2  3  2  1  0 | 30  40  1  11  0  3  1  18  12  2  2  13  8 |

5-ASA: Mesalazine; ADA: Adalimumab; AZA: Azathioprine; CD: Crohn’s disease; F: female; IBD; inflammatory bowel disease; IBDU: inflammatory bowel disease unclassified; IFX: Infliximab; M: male; MMF: Mycophenolate mofetil; MTX: Methotrexate; UC: Ulcerative Colitis; UST: Ustekinumab; VDZ: Vedolizumab.
